# Relationship between feeding modes and infant weight gain in the first month of life

**DOI:** 10.3892/etm.2012.741

**Published:** 2012-10-10

**Authors:** SATOKO EBINA, IKUO KASHIWAKURA

**Affiliations:** 1 Sapporo Medical University, Graduate Course in Midwifery, Chuo-ku, Sapporo 060-8556;; 2 Department of Radiological Life Sciences, Hirosaki University Graduate School of Health Sciences, Hirosaki 036-8564, Japan

**Keywords:** breast-feeding, feeding mode, infant weight gain

## Abstract

Breast-feeding and human milk are beneficial for both mothers and their children. This retrospective study aimed to clarify whether differences in feeding mode influence infant weight gain in the first month of life. We analyzed the pregnancy charts of 422 women who delivered at a birthing center in rural Japan between August 1998 and September 2007. The inclusion criteria were low-risk, full-term pregnancy (duration, 37–42 weeks), spontaneous vaginal delivery, and a healthy infant (1 min Apgar score of ≥8) who underwent a health check-up at 1 month postpartum. The subjects were classified into three groups on the basis of feeding modes: exclusive breast-feeding group (28.9%), mixed-feeding group (55.9%) and exclusive formula-feeding group (15.2%). The weight gain/day was 39.7±9.3 g (range, 18.5–67.4 g), 39.5±9.4 g (range, 13.8–64.5 g) and 39.0±9.5 g (range, 14.4–65.3 g) in the exclusive breast-feeding, mixed-feeding and exclusive formula-feeding groups, respectively. Apart from the rate of maternal smoking, which was lower in the exclusive breast-feeding group, no other significant differences were observed among the three groups. This study revealed that there were no differences in weight gain among infants raised exclusively on breast milk and those raised exclusively on formula milk.

## Introduction

The American Academy of Pediatrics (1) and the World Health Organization (WHO) (2) recommend exclusive breast-feeding for infants until the age of 6 months. A US national survey conducted in 2001 reported that 33% of infants were breast-fed and 17% were exclusively breast-fed until 6 months of age (3). Breast-feeding is the ideal feeding mode for both infants and mothers. Breast-feeding has beneficial effects in infants and mothers, and it plays a crucial role in public health (1). Human milk is species-specific and contains optimal nutrients, growth factors and the immunological components required for infants (4). Breast-feeding has short-term benefits such as the prevention of infectious diseases in infants, including diarrhea (5–8), respiratory tract infection (9–12) and otitis media (13,14). The long-term health benefits of breast-feeding in infants include a lower risk of developing diseases such as obesity (15,16), hypertension (17,18) and type 1 diabetes mellitus (19,20) in adulthood. Gluckman and Hanson (21) proposed the concept of developmental origins of health and disease, which states that unbalanced nutrition in utero and in infancy leads to subsequent disorders.

Body weight is an important index of infant growth and development. Neonates lose weight because of the physiologic extracellular fluid diuresis following their transition from intrauterine to extrauterine life (22). The normal maximal weight loss is 5.5–6.6% of birth weight in optimally exclusively breast-fed infants and occurs between the second and third day of life (23,24). According to the WHO Multicentre Growth Reference Study, a male infant gains 1,200 g/month and a female infant gains 1,000 g/month under optimal conditions. Weight gain in the first month of life in exclusively breast-fed infants is reported to be 18–35 g (25,26). Although studies indicate sufficient evidence regarding breast-feeding, there is no universal agreement among healthcare providers regarding weight gain in breast-fed and formula-fed infants. When weight gain occurs gradually, appropriate support should be provided after determining whether the infant is demonstrating delayed weight gain or a failure to thrive (27–29).

This retrospective study aimed to clarify whether differences in feeding modes influence neonatal weight gain in the first month of life.

## Materials and methods

### Study population

This retrospective study involved the review of pregnancy charts of 422 women who delivered at a birthing center in Aomori Prefecture, Japan. The study complied with the principles of the Declaration of Helsinki (Seoul 2008) and the ethical guidelines for epidemiological research issued by the Ministry of Education, Culture, Sports, Science and Technology as well as the Ministry of Health, Labour and Welfare in Japan (2008). All study data were obtained from the medical records of the subjects and were coded to avoid disclosure of the their identity. We gathered the medical records of 579 women who had a singleton pregnancy and delivered a live infant from August 1998 to September 2007. The inclusion criteria were low-risk, full-term pregnancy (duration, 37–42 weeks), spontaneous vaginal delivery, <500 ml intrapartum blood loss and a healthy infant (1 min Apgar score of ≥8) who underwent a health check-up at 1 month postpartum. Mothers with chronic diseases (e.g., diabetes, hypertension and hyperthyroidism), gestational diabetes, and pregnancy-induced hypertension were excluded from the study. Records with unknown or missing obstetric data were not included. A total of 422 records were finally available for analysis.

### Perinatal parameters

The perinatal parameters extracted from the pregnancy charts were maternal age, parity, smoking status, self-reported prepregnancy weight, prepregnancy body mass index (BMI), gestational weight gain, chronic diseases, delivery mode, duration of pregnancy, infant gender, infant size at birth (weight, height, head circumference and chest circumference), 1 min Apgar score, admission to hospital, and data from the first month health check-up (mother’s weight, feeding mode and infant size).

### Feeding modes

Subjects were divided into three groups on the basis of self-reported feeding mode used by mothers in the first postpartum month: an exclusive breast-feeding group, a mixed-feeding (breast-feeding and formula-feeding) group, and an exclusive formula-feeding group.

### Statistical analysis

Statistical analysis was performed using SPSS software, version 16.0 (SPSS Japan, Inc., Tokyo, Japan) for Windows. Descriptive statistics are indicated as arithmetic mean ± standard deviation. A two-sample t-test, one-way analysis of variance, and Tukey’s honestly significant difference test were performed to determine differences across the three groups. The χ^2^ statistic was used to analyze categorical variables. Univariate analysis was performed using Pearson’s correlation coefficient. A P-value of <0.05 was determined to be indicative of a statistically significant result.

## Results

### Summary of the study population

The maternal parameters of the study population are summarized in Table I. The mean maternal age was 26.6±4.5 years (range, 17–38 years); 38.6% subjects were primiparas and 17.8% were smokers. The mean duration of pregnancy was 39.5±1.2 weeks. The infant parameters of the study population are summarized in Table II. The mean infant birth weight and height were 3,209±370.4 g and 49.6±1.8 cm, respectively; 49.8% infants were male. The mean infant weight and height at 1 month were 4,513.2±451 g and 54.9±1.6 cm, respectively; the mean total weight gain was 1,303.5±325.0 kg, and the weight gain/day was 39.5±9.4 g. The study population was classified into the following three groups on the basis of the feeding mode: breast-feeding (28.9%), mixed-feeding (55.9%) and formula-feeding (15.2%) groups. The overall maternal smoking rate was 17.8%, the smoking rates for the exclusive breast-feeding, mixed feeding, and exclusive formula-feeding groups were 7.4, 20.3 and 28.1%, respectively. The smoking rate was significantly lower in the exclusive breast-feeding group than in the other groups. Except for maternal smoking status, none of the other maternal and infant parameters significantly differed among the three feeding modes.

### Relationship between weight gain and maternal/neonatal parameters

**Figure 1:**
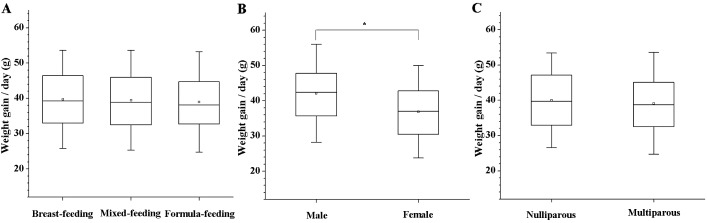
Infant weight gain/day in the first month of life classified on the basis of feeding mode, infant gender, and parity. No significant differences were observed in weight gain/day among (A) the three feeding modes or (C) parity. (B) However, weight gain/day was higher in male infants than in female infants (42.1±9.3 g vs. 36.9±8.8 g; ^*^P<0.05).

Infant weight gain/day in the first month of life was determined for feeding modes, parity and infant gender (Fig. 1). The daily weight gain was 39.7±9.3 g (range, 18.5–67.4 g) in the exclusive breast-feeding group, 39.5±9.4 g (range, 13.8–64.5 g) in the mixed-feeding group, and 39.0±9.5 g (range, 14.4–65.3 g) in the exclusive formula-feeding group. No significant differences in daily weight gain were observed among the feeding modes (Fig. 1A). The daily infant weight gain was significantly higher in the male infants (42.1±9.3 g) than in the female infants (36.9±8.8 g) (Fig. 1B), but did not differ between primiparous and multiparous mothers (Fig. 1C).

Multiple linear regression analysis was used to identify independent variables associated with infant weight gain (Table III). Although the coefficient of determination (R^2^) was low, infant gender, birth weight, birth height, and maternal age were identified as variables that could independently predict the weight gain/day in the first month of life (R^2^= 0.13, P<0.001 by ANOVA).

## Discussion

In Japan, breast-feeding has been promoted since 1989 according to a joint announcement ‘The Ten Steps to Successful Breastfeeding’ released by WHO/UNICEF. According to a national Japanese study in 2005, 42.4% infants aged 1 month, 38.0% aged 3 months, and 34.7% aged 6 months were exclusively breast-fed (30). However, in our retrospective study, 28.9% infants were exclusively breast-fed at 1 month after birth, indicating that the values in our study in Japan were below the national average. The results obtained in the present study were possibly affected by regional characteristics, different attitudes, and/or lack of education on breast-feeding. Moreover, relatively higher birth weights of both male (3,235 g) and female (3,183 g) infants were observed in our study when compared with the average birth weight in Japan (male infants, 3,040 g; female infants, 2,960 g) (31). This finding may be attributed to the low-risk pregnancies, young maternal age, and high rate of multiparity and full-term deliveries in our study. Except for the rate of maternal smoking, which was lower in the exclusive breast-feeding group than in other groups, none of the other maternal and infant parameters assessed in this study significantly differed among the three groups.

According to the WHO Multicenter Growth Reference Study, a male infant gains 1,200 g/month and a female infant gains 1,000 g/month under optimal conditions (25). In the current study, the male infants gained 1,391.1±328.4 g/month (42 g/day) and the female infants gained 1,216.7±297.7 g/month (36.9 g/day). WHO (32) and Dewey (33) reported that male infants gain significantly more weight than female infants. Breast-fed infants weighed significantly less than formula-fed infants at ages 3 months to 2 years; however, no significant differences in weight were observed at age 2–3 months (34–36). The weight gained in the first month after birth did not differ with the feeding modes; this finding was consistent with that of a previous study (24). Exclusively breast-fed infants may gradually gain weight in the first month of life. However, delayed weight gain cannot be disregarded as it may be associated with less than optimal breast-feeding or illness in the infant (e.g., congenital heart disease, neuromuscular disease, infection, or endocrine and metabolic abnormalities). Therefore, infant weight gain assessments must take into account potential illnesses in addition to maternal health, the condition of breast milk, and infant position and latching during breast-feeding. Furthermore, some infants gain less than 20 g/day regardless of the feeding mode. Factors affecting the mother’s and infant’s health after childbirth include abuse, parenting anxiety, and postpartum depression; therefore, the mother’s physical and mental state should be assessed along with the infant’s condition. Continuous support provided to the mother and infant soon after discharge from the hospital is related with improved health of both the mother and her infant.

The present study revealed that weight gain in the first month of life does not significantly differ between exclusively breast-fed infants and exclusively formula-fed infants (Fig. 1). Poor weight gain in infants is not only caused by poor milk intake due to incorrect suckling or insufficient lactation but may also be associated with illness in the infant. Breast-feeding improves the health and development of both the mother and infant. Therefore, assessment of feeding soon after birth is crucial with regard to infant growth and development and prevention of diseases in adulthood. Healthcare providers working with mothers and infants should understand the patterns of growth and development of exclusively breast-fed infants, correctly assess the effectiveness of breast-feeding, and provide continuous support so that both the mother and infant benefit from breast-feeding.

## Figures and Tables

**Table I:** Summary and comparison of maternal parameters classified on the basis of feeding modes.

		Feeding mode group		
Parameters	Total population (n=422)	Breast-fed (n=122)	Mixed-fed (n=236)	Formula-fed (n=64)
Age (years)^a^	26.6±4.5	26.8±4.0	26.6±4.7	26.3±4.9
Duration of pregnancy (weeks)^a^	39.5±1.2	39.5±1.1	39.6±1.2	39.2±1.1^b^
Primiparous^c^	163 (38.6)	44 (36.1)	96 (40.7)	23 (35.9)
Smokers^c^	75 (17.8)	9 (7.4)	48 (20.3)	18 (28.1)^d^
Prepregnancy weight (kg)^a^	53.3±8.4	52.4±6.8	53.7±9.3	54.0±7.5
Prepregnancy BMI^a^	21.2±3.2	20.8±2.4	21.4±3.5	21.4±3.1
Delivery weight	65.2±8.7	64.2±7.4	65.7±9.2	65.3±8.9
Gestational weight gain (kg)^a^	11.8±4.0	11.7±3.4	12.0±4.3	11.3±3.9
Weight at 1 month postpartum (kg)^a^	57.9±8.3	57.0±7.2	58.4±8.9	57.6±8.1
Postpartum weight loss (kg)^a^	7.3±2.3	7.2±2.1	7.3±2.3	7.7±2.6

Values are presented as means ± SD. BMI, body mass index [weight in kilograms divided by the square of the height in meters (kg/m^2^)];

a One-way analysis of variance,

b P<0.05.

c Presented as number and percent and analyzed with the χ^2^,

d P<0.01.

**Table II:** Summary and comparison of infant parameters classified on the basis of feeding modes.

		Feeding modes group		
Parameters	Total population (n=422)	Breast-fed (n=122)	Mixed-fed (n=236)	Formula-fed (n=64)
Male^a^	210 (49.8)	52 (42.6)	126 (53.4)	32 (50.0)
1 min Apgar score^b^	9.8±0.4	9.8±0.4	9.8±0.4	9.7±0.5
Birth				
Weight (g)^b^	3,209.0±370.4	3,203.6±319.6	3,222.3±402.5	3,170.6±337.6
Height (cm)^b^	49.6±1.8	49.6±1.6	49.7±2.0	49.5±1.7
Head circumference (cm)^b^	33.4±1.3	33.3±1.3	33.5±1.3	33.3±1.2
Chest circumference (cm)^b^	31.7±1.6	31.6±1.5	31.8±1.6	31.3±1.7
At 1 month of life				
Weight (g)^b^	4,513.2±451.8	4,504.2±447.8	4,525.3±470.3	4,485.5±390.4
Height (cm)^b^	54.9±1.6	54.8±1.5	55.0±1.8	54.8±1.3
Head circumference (cm)^b^	37.1±1.1	37.2±1.0	37.2±1.1	37.4±1.4
Chest circumference (cm)^b^	37.1±1.4	37.1±1.3	37.0±1.4	37.1±1.2

Values are presented as mean ± SD.

a Presented as number and percent and analyzed with the χ^2^, NS.

b One-way analysis of variance, NS; NS, not significant.

**Table III:** Multiple linear regression analysis for the association between infant weight gain/day and maternal/infant parameters.

Explanatory variable	Standardized partial regression coefficient (β)
Infant gender	−0.276^a^
Birth weight	−0.378^a^
Birth height	0.255^b^
Maternal age	−0.119^c^

Coefficient of determination, R^2^=0.13.

a P<0.001,

b P<0.01,

c P<0.05.

## References

[1] American Academy of Pediatrics (2005). Breastfeeding and the use of human milk. Pediatrics.

[2] WHO (2002). Global Strategy for Infant and Young Child Feeding 55th World Health Assembly.

[3] Ryan AS, Wenjun Z, Acosta A (2002). Breastfeeding continues to increase into the new millennium. Pediatrics.

[4] Prentice A (1996). Constituents of human milk. Food and Nutrition Bulletin.

[5] Howie PW, Forsyth JS, Ogston SA, Clark A, Florey CD (1990). Protective effect of breast feeding against infection. BMJ.

[6] Clemens J, Rao M, Ahmed F (1993). Breast-feeding and the risk of life-threatening rotavirus diarrhea: prevention or postponement?. Pediatrics.

[7] Lopez-Alarcon M, Villalpando S, Fajardo A (1997). Breast-feeding lowers the frequency and duration of acute respiratory infection and diarrhea in infants under six months of age. J Nutr.

[8] Bhandari N, Bahl R, Mazumdar S, Martines J, Black RE, Bhan MK, Infant Feeding Study Group (2003). Effect of community-based promotion of exclusive breastfeeding on diarrhoeal illness and growth: a cluster randomized controlled trial. Lancet.

[9] Blaymore Bier J, Oliver T, Ferguson A, Vohr BR (2002). Human milk reduces outpatient upper respiratory symptoms in premature infants during their first year of life. J Perinatol.

[10] Bachrach VR, Schwarz E, Bachrach LR (2003). Breastfeeding and the risk of hospitalization for respiratory disease in infancy: a meta-analysis. Arch Pediatr Adolesc Med.

[11] Chantry CJ, Howard CR, Auinger P (2006). Full breastfeeding duration and associated decrease in respiratory tract infection in US children. Pediatrics.

[12] Wright AL, Holberg CJ, Martinez FD, Morgan WJ, Taussig LM (1989). Breast feeding and lower respiratory tract illness in the first year of life. Group Health Medical Associates. BMJ.

[13] Owen MJ, Baldwin CD, Swank PR, Pannu AK, Johnson DL, Howie VM (1993). Relation of infant feeding practices, cigarette smoke exposure, and group child care to the onset and duration of otitis media with effusion in the first two years of life. J Pediatr.

[14] Aniansson G, Alm B, Andersson B (1994). A prospective cohort study on breast-feeding and otitis media in Swedish infants. Pediatr Infect Dis J.

[15] Von Kries R, Koletzko B, Sauerwald T (1999). Breast feeding and obesity: cross sectional study. BMJ.

[16] Owen CG, Martin RM, Whincup PH, Smith GD, Cook DG (2005). Effect of infant feeding on the risk of obesity across the life course: a quantitative review of published evidence. Pediatrics.

[17] Singhal A, Cole TJ, Lucas A (2001). Early nutrition in preterm infants and later blood pressure: two cohorts after randomised trials. Lancet.

[18] Horta BL, Bahl R, Martinés JC, Victora CG (2007). Evidence on the long-term effects of breastfeeding: Systematic reviews and meta-analysis.

[19] Virtanen SM, Räsänen L, Aro A (1991). Infant feeding in Finnish children less than 7 yr of age with newly diagnosed IDDM. Childhood Diabetes in Finland Study Group. Diabetes Care.

[20] Gerstein HC (1994). Cow’s milk exposure and type I diabetes mellitus. A critical overview of the clinical literature. Diabetes Care.

[21] Gluckman P, Hanson M (2006). The conceptual basis for the developmental origins of health and disease. Developmental Origins of Health and Disease.

[22] Cunningham FG, Leveno KJ, Bloom SL, Cunningham FG, Leveno KJ, Bloom S (2010). The newborn infant. Williams Obstetrics.

[23] Rodríguez G, Ventura P, Samper MP, Moreno L, Sarría A, Pérez-González JM (2000). Changes in body composition during the initial hours of life in breast-fed healthy term newborns. Biol Neonate.

[24] MacDonald PD, Ross SR, Grant L, Young D (2003). Neonatal weight loss in breast and formula fed infants. Arch Dis Child Fetal Neonatal Ed.

[25] WHO (1999). Evidence for the ten steps to successful breastfeeding.

[26] UNICEF-World Health Organization (1993). Breastfeeding Management and Promotion in a Baby-Friendly Hospital: an 18-hour course for maternity staff.

[27] Mohrbancher N, Stock JMA (2003). The Breastfeeding Answer Book.

[28] Lawrence RA, Lawrence RM (2005). Breastfeeding; A Guide for the Medical Profession.

[29] Regina E, Giugliani J, Mannel R, Martens P, Walker M (2007). Slow weight gain and failure to thrive. Core Curriculum for Lactation Consultant Practice.

[30] Ministry of Health, Labour and Welfare (http://www.mhlw.go.jp/) Japan Summary of the results of a nutrition survey in infants in FY2005, Breast-feeding division (updated 2006 Jun 29). http://www.mhlw.go.jp/.

[31] (2011). Health, Labour and Welfare Statistics Association. Journal of Health and Welfare Statistics.

[32] World Health Organization The WHO Multicentre Growth Reference Study. http://www.who.int/childgrowth/mgrs/en/.

[33] Dewey KG (2001). Nutrition, growth, and complementary feeding of the breastfed infant. Pediatr Clin North Am.

[34] Dewey KG, Heinig MJ, Nommsen LA, Peerson JM, Lönnerdal B (1992). Growth of breast-fed and formula-fed infants from 0 to 18 months: the DARLING Study. Pediatrics.

[35] Kramer MS, Guo T, Platt RW, Vanilovich I (2004). The promotion of breastfeeding intervention trials study group. Feeding effects on growth during infancy. J Pediatr.

[36] Dewey KG (2009). Infant feeding and growth. Adv Exp Med Biol.

